# Effects of Fluorescence‐Activated Cell Sorting on Boar Sperm Motility Subpopulations and In Vitro Embryo Development

**DOI:** 10.1002/mrd.70123

**Published:** 2026-06-07

**Authors:** Isabel Rodriguez, Tyler Weide, Caleigh E. Grote, Alexandra Keller, Catarina Bittencourt, Emily Schoelerman, Ian Shofner, Aileen F. Keating, Bethany K. Redel, Karl Kerns

**Affiliations:** ^1^ Department of Animal Science Iowa State University Ames Iowa USA; ^2^ Interdepartmental Genetics and Genomics Iowa State University Ames Iowa USA; ^3^ USDA‐ARS, Plant Genetics Research Unit Columbia Missouri USA

**Keywords:** blastocyst development, fertilization percentage, in vitro fertilization, sperm sorting

## Abstract

Flow cytometric sorting can be used to select sperm subpopulations with defined physiological or molecular traits; however, the impact of sorting on sperm function and subsequent embryo development in the porcine model remains unresolved. The objective of this study was to evaluate the effect of fluorescence‐activated cell sorting (FACS) on boar sperm motility and developmental competence following in vitro fertilization (IVF). Pooled semen from boars was divided into sorted and unsorted (control) treatments and analyzed for motility and kinematic parameters using computer‐aided sperm analysis (CASA) with both population‐level (conventional, population‐averaged) metrics and single‐cell track data collected before and after sorting. Oocytes were fertilized with either treatment, and cleavage (≥ 2‐cell stage) and blastocyst development were evaluated. Cleavage and blastocyst percentages were reduced in the sorted (48.8% ± 9.9% and 8.5% ± 4.2%) compared with control (92.2% ± 1.5% and 28.4% ± 2.8%, respectively; *p* < 0.001) group. Population‐level CASA revealed statistically significant but biologically modest changes in motility and kinematic parameters following sperm sorting. In contrast, single‐cell CASA coupled with *k*‐means clustering identified four distinct motility subpopulations and demonstrated that sorting redistributed existing motility states, characterized by depletion of fast, progressive sperm and enrichment of less energetically demanding populations. These findings suggest that FACS alters the composition of sperm motility subpopulations, alongside a marked reduction in post‐fertilization developmental competence in porcine IVF.

## Graphical abstract

Effects of fluorescence‐activated sperm sorting on IVF outcomes and motility subpopulations. Distinct boar ejaculates were either used directly for in vitro fertilization (IVF; control, unsorted) or processed by fluorescence‐activated cell sorting to generate a sorted sperm population. IVF outcomes demonstrated that oocytes fertilized with unsorted sperm had higher cleavage and blastocyst development percentages compared with those fertilized using sorted sperm. In parallel, both sorted and unsorted sperm samples were evaluated using computer‐assisted semen analysis, including single‐cell motility analysis followed by unsupervised *k*‐means clustering. Four sperm motility subpopulations were identified, and sorting redistributed existing sperm subpopulations rather than generating novel phenotypes.

Created in BioRender. Kerns, K. (2026) https://BioRender.com/c0g162g.

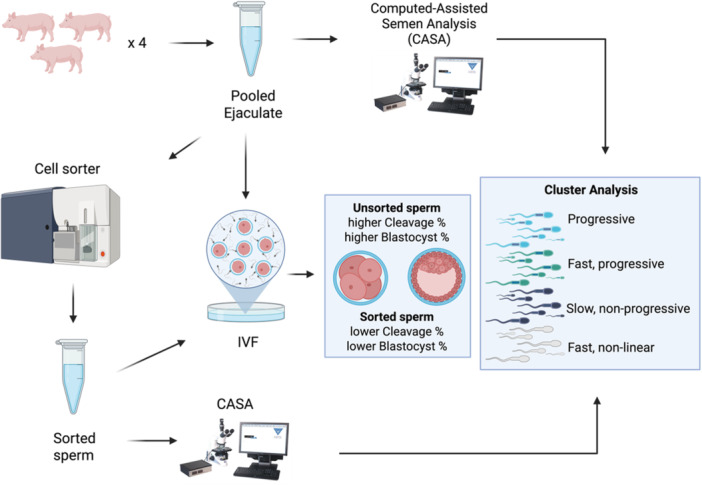

## 1 Introduction

Assisted reproductive technologies (ART) have become essential tools for improving reproductive efficiency and genetic progress in livestock species. Among the reproductive technologies currently used, in vitro fertilization (IVF) enables controlled evaluation of gametes and embryonic development under standardized conditions. Despite meaningful advancements in porcine IVF protocols, fertilization efficiency and development rates remain highly variable, with an overall reduced developmental competency compared with in vivo*‐*derived embryos (Bauer et al. 2010; Macháty et al. 1998). This variability potentially reflects the complexity of gamete physiology, particularly sperm heterogeneity and its influence on fertilization potential. Spermatozoa from a single ejaculate represent a heterogeneous population differing in motility, morphology, and membrane composition (Pitnick et al. 2008). In boars, additional sources of ejaculate variation include genotype (Knecht et al. 2017), seasonal fluctuations in ejaculate volume and total sperm output (Snoj et al. 2013), and breed‐related differences associated with artificial insemination efficiency (Górski et al. 2017). Such variability among sperm subpopulations with distinct functional and fertilizing capacities underscores the need for techniques that can isolate and utilize the most competent sperm for IVF.

Fluorescence‐activated cell sorting (FACS) has emerged as a powerful tool for selecting desired sperm subpopulations. This technology utilizes flow cytometry to separate cells based on fluorescent labeling, allowing discrimination of sperm populations based on specific physiological or molecular characteristics. The earliest application of flow cytometric sorting in reproduction was performed on vole (*Microtus oregoni*) sperm, where X‐ and O‐sperm nuclei were successfully separated based on DNA content differences (Pinkel et al. 1982). This approach was later adapted for use in live mammalian sperm through the introduction of Hoechst 33342 as a vital DNA stain (Morrell et al. 1988), ultimately leading to the development of commercial sex‐sorting applications widely implemented in cattle (Cran et al. 1993). More recently, FACS has been used to isolate sperm based on molecular biomarkers, such as capacitation (Castro et al. 2024) and mitochondrial activity (Sousa et al. 2011). Although flow cytometric sorting has been proven effective for sex selection with bull sperm, concerns remain regarding its potential impact on sperm structure and function. The physical and biochemical stressors introduced to the sperm cells while sorting, such as high pressure (Suh et al. 2005) and shear forces from fluidics (Parast et al. 2023) may influence sperm membrane stability (Mocé et al. 2006), capacitation onset (Bucci et al. 2012), motility (Steele et al. 2020), and morphology (Camara Pirez et al. 2020). Additionally, physiological disruptions such as reduced chromatin integrity (Boe‐Hansen et al. 2005) and increased reactive oxygen species (Balao da Silva et al. 2016) have been observed. Despite these concerns, limited data exist on how the FACS process itself influences boar sperm quality and subsequent embryo development. Porcine IVF using flow cytometrically sex‐sorted sperm has been demonstrated (Abeydeera et al. 1998; Bathgate et al. 2007; Rath et al. 1999); however, those studies utilized substantially lower sperm:oocyte ratios (35–75 sperm per oocyte) than standard porcine IVF protocols, primarily due to limited sorting throughput at the time, and were not designed to evaluate sorting‐associated effects on sperm functional heterogeneity.

Conventional semen analysis provides useful population‐averaged measures of sperm motility and kinematics; however, these metrics often obscure biologically important heterogeneity within sperm populations (Henning et al. 2013). Fertilization competence is increasingly recognized to reside within discrete sperm subpopulations that differ in energetic state and metabolism (Abruzzese et al. 2024), membrane organization (Gadella 2008), and capacitation dynamics (Kerns et al. 2018), rather than in bulk motility alone. While porcine IVF has been performed using flow cytometrically sex‐sorted sperm (Abeydeera et al. 1998; Bathgate et al. 2007; Rath et al. 1999), to our knowledge, no studies have evaluated the effects of FACS‐based sperm processing on boar sperm motility subpopulations at the single‐cell level under standard porcine IVF sperm concentrations, or how those subpopulation shifts relate to cleavage and blastocyst developmental competence.

Despite the routine use of flow cytometric sorting in livestock sex‐selection, the impact of the sorting process itself on boar sperm function and porcine IVF competence (independent of any sex‐ or biomarker‐based selection) has not been systematically characterized at standard porcine IVF conditions. Because FACS uniquely combines the throughput, sensitivity, and live‐cell compatibility required to physically isolate sperm subpopulations for downstream comparative omic analysis, defining its baseline effects on porcine sperm motility structure and embryonic development is a prerequisite for interpreting any study that uses FACS to recover discrete sperm populations.

The objective of this study was thus to evaluate the effect of FACS on boar sperm function and early embryonic development under standard porcine IVF conditions, independent of sex‐selection. Cleavage and blastocyst development were compared between oocytes fertilized with sorted and unsorted sperm. In parallel, sperm motility and kinematic behavior were evaluated at both the population and single‐cell levels before and after sorting using computer‐aided sperm analysis (CASA).

## 2 Materials and Methods

### 2.1 Experimental Design

Two treatments were evaluated, with four biological replicates used for IVF experiments and an additional six biological replicates used for sperm‐only analysis (*n* = 10 total). Treatments consisted of a sorted sperm sample (sorted) and an unsorted sample (control) used for IVF. For each replicate, a pool of boar semen was created by combining ejaculates from three boars (based on equal sperm cell count; one‐third contribution from each boar). Each pooled sample was then divided between the two treatments. Sperm motility and kinematics for both treatments were analyzed using CASA prior to fertilization, and for the sorted group, motility and kinematics were additionally evaluated before and after sorting. In vitro fertilization was performed to compare cleavage success and subsequent blastocyst development between the treatments. Cleavage percentage was calculated as the proportion of metaphase II (MII) oocytes that had reached the ≥ 2‐cell stage relative to the total number of MII oocytes inseminated per well and was assessed approximately 48 hours post‐fertilization (hpf). Blastocyst percentage was calculated as the proportion of early, expanded, and hatching blastocysts relative to the number of cleaved embryos and assessed approximately 144 hpf. Blastocyst efficiency was calculated as the proportion of early, expanded, and hatching blastocysts relative to the number of MII oocytes placed in the fertilization droplet and was assessed at 144 hpf.

### 2.2 Semen Collection and Processing

Commercial Duroc boars of the same relative age from a private boar stud were utilized in this study. For IVF experiments, semen from 12 unique boars was allocated to four pools (three boars per pool; *n* = 4). For motility analyses, these 12 boars comprised the first four pools, and 18 additional boars were included to generate six additional pools (three boars per pool; total *n* = 10). The semen used was not specifically collected for this study; rather, they were an excess of standard production at the facility. Boars were individually housed and collected once weekly via the double‐gloved hand technique. Only ejaculates exhibiting > 80% total motility and morphology were processed further. Immediately after collection, semen was extended a total of 5 times using Preserve Xtreme extender (GENEPRO, Madison, Wisconsin, United States), ensuring the extender was within 2°C of the semen temperature. Samples were transported to Iowa State University on the same day of processing. Upon arrival, the temperature of the samples was recorded, and they were stored at 17°C until day of use, which was 3 days post‐arrival. For each replicate, semen from three unique boars was pooled to account for ejaculate and boar variation. Pools were assembled by contributing equal sperm numbers from each boar (one‐third per boar), and the final pooled working concentration was 30 million cells/mL. Sorted and unsorted treatment aliquots were drawn from this single pooled stock to ensure both treatments originated from identical starting material.

## 3 Flow Cytometric Sorting

### 3.1 Media

#### 3.1.1 Porcine Non‐Capacitation Media (pNCM)

Non‐capacitation media containing NaCl, KCl, NaH2PO_4_, Na lactate, MgCl_2_−6H_2_O, TL‐HEPES, Na‐pyruvate, sorbitol, gentamicin, penicillin G, polyvinyl alcohol (PVA), and glucose were prepared in 1000 mL of deionized water (ddH_2_O), as previously described (Kerns et al. 2018). Vacuum filtration of this solution was performed into an appropriate container, and the pH of the solution was adjusted to 7.20 ± 0.02 and stored at 4°C until use.

### 3.2 FACS Catch Media

AndroStar Premium long‐term extender (LTX) manufactured by Minitube was used as FACS catch media. The solution was prepared and handled according to the manufacturer's guidelines. LTX was supplemented with 1% BSA (Bovine Serum Albumin) to allow for pelleting after centrifugation (Weide et al. 2026).

### 3.3 Reagents

Hoechst 33342 (H33342; Calbiochem, 382065, San Diego, CA, USA) was reconstituted with ddH_2_O to a stock solution of 18 mM.

### 3.4 Flow Cytometry Probe Staining and Incubation

For FACS, sperm concentration was calculated from CASA measurements to adjust to 30 million cells in 600 µL of pNCM and stained with 18 µM H33342 (1:1000 stock). Samples were covered and incubated at room temperature for 30 min, then centrifuged at 500 × g for 4 min. The supernatant was removed, and sperm were resuspended in 1200 µL of room temperature pNCM before sorting.

### 3.5 Fluorescence Activated Cell Sorting

Cell sorting was performed using the BD FACSMelody Cell Sorter (BD Life Sciences, San Jose, CA, USA). The instrument was equipped with three spatially separated excitation lasers: 405 nm violet laser (40 mW), 488 nm blue laser (20 mW), and 640 nm red laser (40 mW). The fluidics system utilized a quartz cuvette flow cell with gel‐coupled optics for optimal light collection and resolution. Sheath fluid consisted of 1× Gibco Phosphate‐Buffered Saline Solution at pH 7.20. A 100 µm nozzle was used to ensure gentle handling of sperm cells. Flow speed was set to 5–10 (no units, BD FACSMelody flow setting) to maintain event rate of 10,000 events/s. Agitation was set at 200 rpm to keep cells suspended. Sorted populations were collected in 5.0 mL polystyrene tubes to limit cell adhesion to the tube and minimize cell loss. The photomultiplier tube settings for the H33342 gated population were set at 55.

The sorting process used BD FACSChorus Software (v1.4.3.0), which provided automated setup and optimization of droplet breakoff, stream alignment, and sort delay using BD FACS Accudrop beads. Hoechst 33342 was used solely as a vital DNA stain to identify sperm cells from debris and doublets; no X/Y bivariate gating was applied. Gating was performed consecutively, beginning with forward scatter (FSC)/side scatter (SSC) to identify sperm and exclude debris, followed by exclusion of doublets using FSC‐W vs. FSC‐H. From the single‐cell population, Hoechst 33342‐positive cells were selected (Supplemental Figures 1 and 2).

A bulk sort was performed for all four sorting positions, collecting approximately 1.296 × 10^6^ cells per 5.0 mL polystyrene tube containing 500 µL of AndroStar Premium long‐term extender (LTX; Minitube, Tiefenbach, Germany) supplemented with 1% BSA as a catch media (4 tubes total). Sorted samples were centrifuged at 500 × g for 10 min in a swinging hinge centrifuge, the supernatant removed, and all fractions recombined into a single 1.5 mL microcentrifuge tube. The recombined fractions were then centrifuged at 500 × g for 4 min, supernatant removed, resuspended with 100 µL of LTX only (no BSA). Following sorting, samples were reassessed by CASA for post‐sorting motility.

### 3.6 Semen Preparation for IVF (Sorted and Unsorted)

Preparation of the sorted semen sample was executed as described above following flow cytometric sorting, post‐sorting CASA assessment. Modified Tris‐buffered medium (mTBM) sealed from ambient air was removed from the 5% CO_2_ incubator and allowed to reach room temperature prior to use to prevent thermal shock to the sperm. A 150 μL aliquot was added to 1 mL of mTBM and centrifuged in a swinging‐hinge rotor centrifuge at 500 × g for 5 min. The supernatant was discarded and the pellet was resuspended in 1 mL fresh mTBM; this wash step was repeated twice. Sperm concentration was then determined by CASA, both samples (sorted and unsorted) were diluted in mTBM to reach a final concentration of 0.5 × 10^6^ cells/mL.

### 3.7 Oocyte Handling, Fertilization, and Culture Media

TL‐HEPES handling solution, oocyte maturation medium, oocyte manipulation medium, MU4 culture medium, and mTBM were prepared and stored following previously described procedures (Redel et al. 2019). To minimize evaporation during incubation, all IVF droplets were overlaid with NidOil (NidaCon International AB, Gothenburg, Sweden).

### 3.8 In Vitro Fertilization

Gilt ovaries were collected at a nearby abattoir and transported to the laboratory. Upon arrival, ovaries were rinsed with warm saline solution kept at 38.5°C and follicles measuring 3–5 mm in diameter were aspirated using an 18‐gauge needle connected to a vacuum pump system. The follicular aspirate was kept warm (38.5°C) while a pellet formed and then washed twice with warm TL‐HEPES (38.5°C). Oocytes surrounded by at least two compact layers of cumulus cells were selected for in vitro maturation. Selected cumulus‐oocyte complexes (COCs) were cultured in TCM‐199‐based maturation medium supplemented with 0.57 mM l‐cysteine, 10 ng/mL of EGF and FSH, 0.5 μg/mL of LH (Redel et al. 2019), 40 ng/mL of FGF, and 20 ng/mL of IGF and LIF (Yuan et al. 2017). The oocytes were incubated at 38.5°C in 5% CO_2_ for 40–44 h. Following maturation, cumulus cells were removed by gentle pipetting in 0.1% (w/v) hyaluronidase for 30 s–1 min. Oocytes displaying an extruded polar body were selected for IVF and transferred in groups of 35–40 into 50 μL droplets of mTBM overlaid with 3 mL of NidOil. The maturation rate, defined as the proportion of cultured COCs reaching MII after 40–44 h of in vitro maturation (IVM), was recorded for each IVF replicate as a quality control metric for the IVM step. The final sperm concentration for both treatments (sorted and unsorted) was adjusted to 0.5 × 10^6^ cells/mL and 50 μL of sperm was introduced into each mTBM droplet for a final concentration of 0.25 × 10^6^ cells/mL. Gametes were co‐incubated for 4 h at 38.5°C under 5% CO_2_. Presumptive zygotes were subsequently washed and cultured in 4‐well Nunc dishes containing 500 μL MU4 overlaid with 250 μL of NidOil. The cells were maintained at 38.5° C in 5% O_2_, 5% CO_2_, and 90% N_2_ for 6 days. Cleavage percentage was assessed approximately 48 hpf, and blastocyst percentage and blastocyst efficiency were assessed approximately 144 hpf. Because only MII oocytes were transferred to the fertilization droplet, the number of MII oocytes inseminated per well represents both the total oocytes placed in IVF culture and the number of presumptive zygotes; cleavage and blastocyst denominators are therefore equivalent under either framing.

### 3.9 Computer‐Aided Sperm Analysis

For concentration and motility, samples (described above) were warmed at 37°C for 10 min before being evaluated using CASA. Samples were gently mixed thoroughly to ensure even distribution of cells and 3.5 µL of each aliquot was loaded onto a 20 µm disposable counting chamber from Minitube (Minitüb GmbH, Tiefenbach, Germany). Concentration and motility were assessed using a Zeiss Axioscope 5 microscope (Carl Zeiss Microscopy LLC, Oberkochen, Germany) fitted with a Basler ace ac2440‐75uc camera (Basler AG, Ahrensburg, Germany) and a 10×/0.25 A‐Plan objective lens using Minitube AndroVision software (Reference Module: 12,500/1000, Tiefenbach, Germany). The condenser with aperture diaphragm was set at 1, and the six‐position filter wheel was set at 2. Videos were acquired at 60 frames per second with a 0.5‐s capture duration per field. For each sample, a minimum of 600 sperm cells were analyzed across four non‐overlapping fields. Motility classification was performed using AndroVision software decision‐tree thresholds. Sperm were classified as immotile when the amplitude of lateral head displacement (ALH) was < 1.00 µm and curvilinear velocity (VCL) was < 24.0 µm/s, or when head activity coefficient (HAC) was < 0.03. Motile sperm were further subdivided, with local motility defined as straight‐line velocity (VSL) < 24.0 µm/s and VCL < 48.0 µm/s. Progressive motility included sperm exhibiting either circular motility, defined by a radius > 10.0 µm and < 30.0 µm with rotation > 0.70, or forward motility characterized as slow motility (VCL < 120.0 µm/s) or fast motility when slow motility criteria were not met. All samples were used at a standardized concentration of 30 million cells/mL prior to CASA loading, applied identically across treatments and replicates to minimize concentration‐dependent collision artifacts and tracking inaccuracy.

### 3.10 Single‐Cell CASA Analysis and Motility Subpopulation Identification

Single‐cell CASA data were extracted from individual sperm tracks and included average path velocity (VAP), linearity (LIN), ALH, and beat‐cross frequency (BCF). Prior to clustering, all variables were standardized using *z*‐score normalization. Unsupervised *k*‐means clustering (*k* = 4; nstart = 50) was applied to identify motility‐based sperm subpopulations, consistent with previous applications of cluster analysis to characterize distinct boar sperm motility patterns and to emphasize the dominant, biologically interpretable motility phenotypes observed in single‐cell CASA clustering frameworks (Henning et al. 2013). Clusters were biologically annotated post hoc on relative differences in velocity and trajectory parameters, resulting in classifications of fast, non‐linear; fast, progressive; progressive; and slow, non‐progressive sperm. These annotations were used consistently across downstream analysis. This *k*‐selection was intended to capture dominant motility states while avoiding over‐partitioning into low‐frequency clusters reported in previous hierarchical approaches.

### 3.11 Statistical Analysis

All statistical analyses were performed using R software version 4.5.1 (R Core Team, 2025). The experimental unit in all analyses was the biological semen pool; for IVF, a single fertilization droplet was inseminated per treatment per replicate. Cleavage percentage, blastocyst percentages, and blastocyst efficiency were analyzed using generalized linear mixed models (GLMM) with a binomial distribution and logit link function in the lme4 package (Bates et al. 2015). Treatment (sorted vs. control) was included as a fixed effect, and replicate (*n* = 4 for IVF, *n* = 10 for motility analysis) was included as a random effect to account for variation among biological replicates. Estimated marginal means were back transformed to the response scale and reported as predicted percentages. Statistical significance for IVF outcomes was defined as *p* < 0.05.

Sperm motility parameters obtained from CASA were analyzed using paired statistical approaches to account for repeated measurements within biological replicates. Differences between unsorted and sorted sperm were evaluated using paired *t*‐tests, and raw *p*‐values were adjusted for multiple comparisons using the Benjamini–Hochberg false discovery rate (FDR) correction (Benjamini and Hochberg 1995), with statistical significance defined as FDR‐adjusted *p* < 0.05.

Single‐cell CASA kinematic data were analyzed using unsupervised *k*‐means clustering (*k* = 4) following standardization of selected kinematic parameters (VAP, LIN, ALH, and BCF) to identify motility‐based sperm subpopulations, as previously described by Henning et al. (2013). These parameters were selected to represent complementary and minimally collinear descriptors of sperm velocity, trajectory linearity, lateral head displacement, and flagellar beat dynamics. To minimize treatment‐driven bias, datasets were randomly balanced within biological replicates prior to clustering. Differences in cluster distributions between treatments were evaluated using Chi‐square tests at the cell level and linear mixed‐effects models at the replicate level, with treatment, cluster identity, and their interaction included as fixed effects and replicate included as a random effect. Replicate‐level mixed‐effects models were used to avoid pseudoreplication arising from cell‐level clustering analyses. Dimensionality reduction using t‐distributed stochastic neighbor embedding (t‐SNE) was performed for visualization purposes only using the same standardized kinematic variables to facilitate comparison with clustering results.

## 4 Results

**FIGURE 1:**
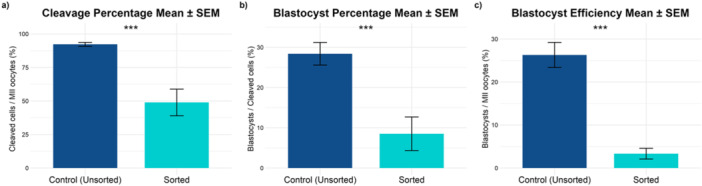
Cleavage and blastocyst percentages of oocytes following in vitro fertilization using sorted vs. unsorted boar sperm. (a) Mean (±SEM) cleavage percentage, calculated as the proportion of MII oocytes that cleaved (≥ 2‐cell stage) following IVF with either sorted sperm or unsorted sperm (control). Cleavage percentage was evaluated approximately 48 hpf. (b) Mean (±SEM) blastocyst percentage, calculated as the proportion of cleaved (≥ 2‐cell stage) embryos that developed to the blastocyst stage after IVF using either sorted sperm or unsorted sperm. (c) Mean (±SEM) blastocyst efficiency, calculated as the proportion MII oocytes that reached the blastocyst stage. Blastocyst percentage and blastocyst efficiency were evaluated approximately 144 hpf. For all panels, each replicate (*n* = 4) represented a pooled semen sample from three boars. Statistical differences are indicated by *** *= p* < 0.001.

Across the four IVF replicates, 58.3% ± 2.2% of cultured COCs reached MII and were selected for insemination. Maturation efficiency was equivalent across treatment‐assigned pools, as oocytes were randomized to treatment after MII selection. Cleavage percentage differed between sorted and unsorted (control) treatments (*p* < 0.001). Oocytes fertilized with unsorted sperm had a higher cleavage percentage (92.2% ± 1.5%) compared to those fertilized with sorted sperm (48.8% ± 9.9%; Figure 1a). The estimated treatment effect (*β* = −2.65 ± 0.36), indicated that sperm sorting decreased the odds of cleavage by approximately 93% relative to control (odds ratio = 0.07). Random variation among replicates was low (0.21). Similarly, blastocyst development among cleaved oocytes was lower in the sorted group compared with the control (*p* < 0.001). The proportion of cleaved oocytes developing to the blastocyst stage was reduced in the sorted (8.5% ± 4.2%) compared with the control sperm group (28.4% ± 2.8%; Figure 1b). The estimated treatment effect for blastocyst development (*β* = −1.66 ± 0.50) indicated that sperm sorting reduced the odds of blastocyst formation of cleaved oocytes by approximately 81% compared with the control (odds ratio = 0.19, *p* < 0.001). Furthermore, evaluation of blastocyst efficiency showed amplified developmental losses. Blastocyst efficiency was higher in the control group (26.3% ± 2.9%) vs. the sorted group (3.4% ± 1.3%; *p* < 0.001; Figure 1c). The estimated treatment effect (*β* = −2.31 ± 0.49) corresponded to an approximately 90% reduction in the odds of any given inseminated MII oocyte reaching the blastocyst stage following sperm sorting (odds ratio = 0.10).

**FIGURE 2:**
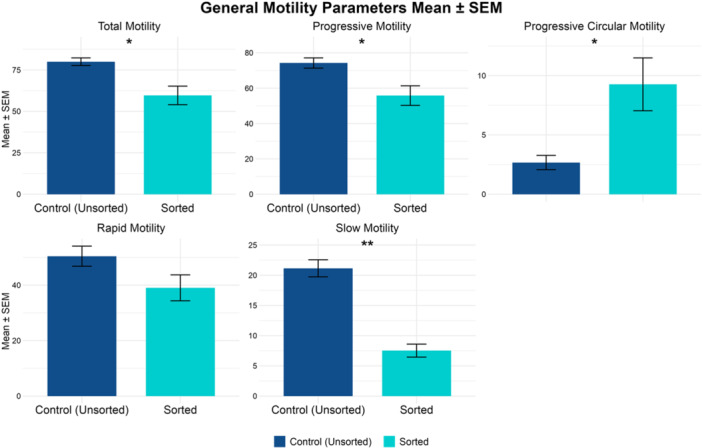
General motility parameters of boar sperm by treatment. Mean (±SEM) values of total motility, progressive motility, progressive circular motility, rapid motility, and slow motility measured using CASA before (control, unsorted) and after sorting (sorted) boar sperm. Detailed statistical results are provided in Supplemental Table 1. The data represent the mean of 10 replicates (*n* = 10), with each replicate consisting of a pool of three different boars. Statistical differences between treatments were assessed using paired *t*‐tests with *p*‐values adjusted for multiple comparisons using Benjamini–Hochberg FDR. Asterisks indicate FDR‐adjusted significance (* = FDR < 0.05, ** = FDR < 0.01).

Motility and kinematic traits in pooled semen samples used for fertilization were measured by CASA before (unsorted) and after sorting (sorted). General motility parameters, including total motility, progressive motility, progressive circular motility, and slow motility, differed between treatments, whereas rapid motility was not altered (Figure 2). Detailed statistical results and *p*‐values are presented in Supplemental Table 1.

**FIGURE 3:**
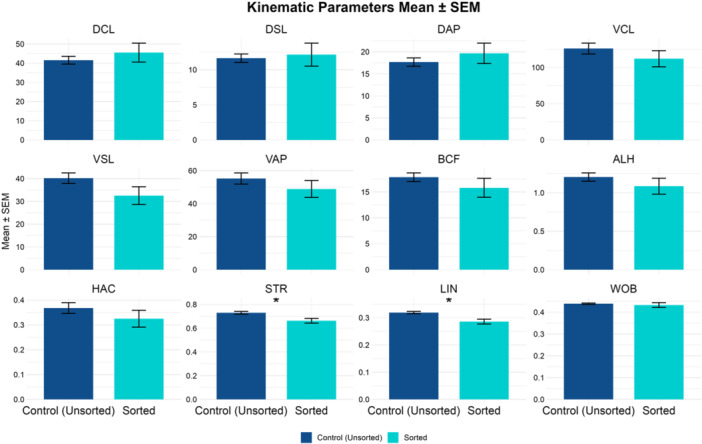
Detailed motility parameters of boar sperm by treatment. Mean (±SEM) for 12 kinematic parameters, including curvilinear distance (DCL), straight line distance (DSL), average path distance (DAP), VCL, VSL, VAP, BCF, ALH, HAC, STR, LIN, and wobble (WOB). Each parameter was measured using CASA before (control, unsorted) and after sorting (sorted) boar sperm for 10 biological replicates (*n* = 10). Detailed statistical results are provided in Supplemental Table 2. Each replicate represented a pooled semen sample from three boars. Statistical differences between treatments were assessed using paired *t*‐tests with *p*‐values adjusted for multiple comparisons using Benjamini–Hochberg FDR. Asterisks indicate FDR‐adjusted significance (* = FDR < 0.05).

Detailed kinematic parameters encompassing distance, velocity, head movement, flagellar activity, and morphometric and trajectory descriptors were further evaluated. Of the 12 parameters assessed, only LIN and straightness (STR) differed between treatments (Figure 3). Detailed statistical results and *p*‐values are presented in Supplemental Table 2.

**FIGURE 4:**
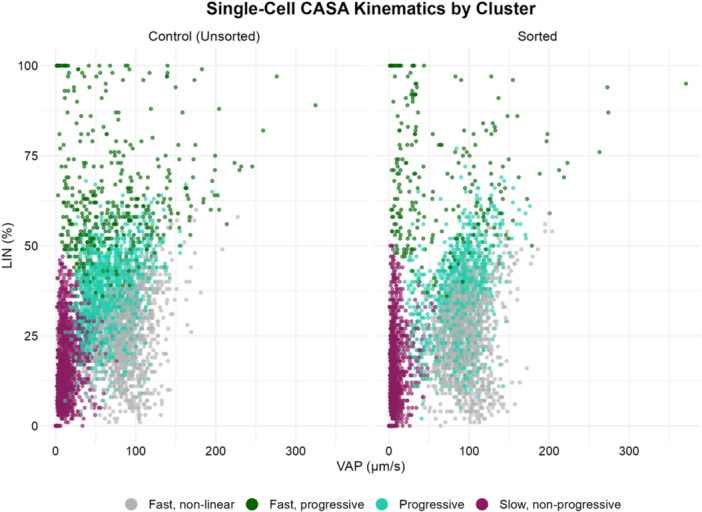
Single‐cell CASA‐based cluster analysis. Single‐cell CASA kinematic parameters, VAP, LIN, ALH, and BCF were subjected to unsupervised *k*‐means clustering (*k* = 4; nstart = 50). Clusters were biologically annotated post hoc on relative velocity and linearity parameters as fast, non‐linear; fast, progressive; progressive; and slow, non‐progressive. Specific kinematic profiles for each cluster are presented in Supplemental Figure 1. Prior to clustering, the dataset was balanced within each replicate (*n* = 10) between treatments for equal cell counts. Each point represents an individual sperm cell.

Single‐cell CASA analysis, followed by *k*‐means clustering (*k* = 4), identified four sperm subpopulations, denoted as fast, non‐linear; fast, progressive; progressive; and slow, non‐progressive phenotypes (Figure 4). Clusters were biologically annotated based on relative differences in velocity and trajectory parameters. The fast, progressive cluster was characterized by high VAP and high LIN, whereas the fast, non‐linear cluster exhibited similarly high velocities but reduced linearity. The progressive cluster displayed intermediate velocity with high linearity, while the slow, non‐progressive cluster was defined by low velocity and limited forward progression (Supplemental Figure 1). Cluster distributions were quantified per replicate (*n* = 10), with a matched number of sperm cells analyzed per replicate between the unsorted and sorted treatments and compared between unsorted and sorted samples.

At the single‐cell level, a Chi‐square test indicated that sperm sorting altered the distribution of motility clusters (*χ* ^2^ = 111.4, df = 3, *p* < 0.0001). Examination of standardized residuals demonstrated depletion of fast, progressive sperm and enrichment of progressive sperm in the sorted relative to control group, whereas fast, non‐linear and slow, non‐progressive clusters had minimal deviations. Collectively, these results indicate that sperm sorting redistributes existing motility subpopulations rather than uniformly altering sperm motility.

**FIGURE 5:**
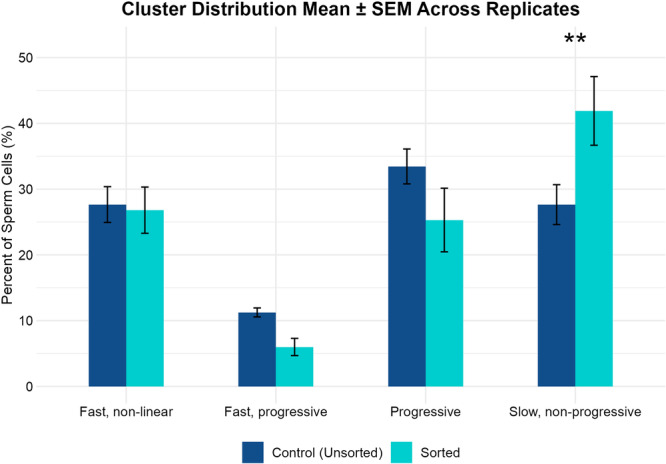
Replicate‐level cluster distribution (mean ± SEM). Bars represent the mean percentage (±SEM) of sperm cells per cluster calculated across replicates (*n* = 10 replicates). Treatment effects on cluster proportions were evaluated using a linear mixed‐effects (LMER) model, with treatment, cluster identity, and their interaction included as fixed effects, and replicate included as a random effect. Model‐based pairwise comparisons between control (unsorted) and sorted samples were performed within each cluster using estimated marginal means. Statistical differences are indicated by ** = FDR < 0.01.

To determine whether these redistribution patterns were consistent across biological replicates, replicate‐level cluster proportions were analyzed using a linear mixed‐effects model with treatment, cluster identity, and their interaction as fixed effects and replicate included as a random intercept. Treatment alone did not affect overall cluster proportions (*p* = 0.86); however, a treatment x cluster interaction was detected for the slow, non‐progressive subpopulation (*p* = 0.026), with sorted samples having a higher proportion of slow, non‐progressive sperm relative to unsorted controls (Figure 5). No treatment x cluster effects were detected for fast, non‐linear, fast, progressive, or progressive clusters, although directional trends were observed.

**FIGURE 6:**
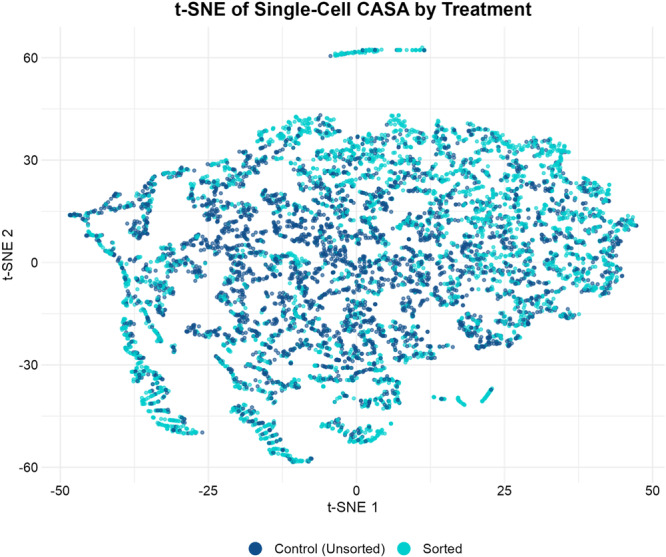
t‐SNE visualization by treatment. A single t‐distributed stochastic neighbor embedding (t‐SNE; perplexity = 30) was generated from single‐cell CASA variables previously described (VAP, LIN, ALH, and BCF) and colored by treatment.

Dimensionality reduction using t‐distributed stochastic neighbor embedding (t‐SNE) was performed to visualize single‐cell CASA data by treatment, as shown in Figure 6. The resulting embedding showed substantial overlap between sorted and unsorted sperm populations, indicating that sorting did not generate novel motility phenotypes but redistributed cells within an existing motility landscape. Visualization of single‐cell CASA by cluster and treatment is presented in Supplemental Figure 2.

## 5 Discussion

The present study was designed as a baseline characterization of how the FACS process itself, independent of sex‐ or biomarker‐based selection, affects boar sperm function and porcine IVF outcomes at standard sperm:oocyte ratios. Our findings demonstrated that flow cytometric sorting of boar sperm reduces both cleavage percentage and blastocyst development following IVF, despite only modest alterations in CASA motility parameters. Although sorting induced detectable changes in motility and kinematic behavior, these changes were not of sufficient magnitude or pattern to account for the pronounced reduction in embryonic development, suggesting that subtle physiological or molecular perturbations induced during the sorting process likely contribute to the observed developmental defects.

Prior porcine studies using flow cytometric sex‐sorting demonstrated the feasibility of IVF and embryo transfer with pre‐sexed sperm, with cleavage rates reported as comparable to or higher than unsorted controls (Abeydeera et al. 1998; Bathgate et al. 2007; Rath et al. 1999). Critically, those studies employed sperm:oocyte ratios of 35–75, substantially lower than the concentrations used in standard porcine IVF protocols (0.25–0.5 × 10^6^ cells/mL; Redel et al. 2019; Yuan et al. 2017), primarily because sorting throughput at the time limited the number of sorted sperm available. In the present study, sorted and unsorted sperm were used at a standard porcine IVF concentration, resulting in approximately 625–714 sperm per oocyte, an order of magnitude greater than in prior sex‐sorting IVF studies. Flow cytometric sorting has been shown to induce capacitation‐like membrane changes in boar sperm, as evidenced by increased capacitation‐associated chlortetracycline (CTC) B‐pattern staining (Bucci et al. 2012), and to alter subsequent capacitation dynamics, including zinc efflux and plasma membrane remodeling (Weide et al. 2026). These sorting‐induced physiological shifts, when combined with higher sperm:oocyte ratios, may amplify polyspermy and compromise post‐fertilization developmental competence, an interaction that could not manifest at the low sperm numbers used in earlier sex‐sorting studies. Whether sorting‐induced capacitation‐like changes interact with sperm:oocyte ratio to increase polyspermy under standard porcine IVF conditions warrants direct investigation in future studies.

When evaluated as overall technical efficiency (blastocyst produced per inseminated oocyte), the developmental decline associated with sorting was further amplified, indicating that FACS‐associated losses are not confined to a single embryonic transition but compound across fertilization, cleavage, and post‐cleavage development. The reduction in cleavage and blastocyst development observed herein is directionally consistent with early bovine IVF studies using sex‐sorted sperm, although the magnitude and manifestation of these effects appear to differ across species and experimental systems. In cattle, lower cleavage and blastocyst rates following IVF with sex‐sorted sperm compared with unsorted controls were reported, accompanied by blastocyst ultrastructural abnormalities such as mitochondrial immaturity and widened nuclear membranes, while also documenting substantial bull‐dependent variation (Palma et al. 2008). Subsequent work demonstrated that while fertilization rates were not always impaired, blastocyst development was reduced in a bull‐dependent manner, suggesting that sorting primarily impacts post‐fertilization developmental competence (Inaba et al. 2016). More recent bovine findings indicate that embryos generated using sex‐sorted sperm exhibit an increased probability of arrest at the zygote and 4‐cell stages, resulting in reduced blastocyst formation compared with embryos produced using conventional sperm (Steele et al. 2020). Together, these findings support the concept that sperm sorting can compromise early embryonic development, while also highlighting important species‐ and sire‐dependent sensitivities. In the present study, pooled semen was intentionally used to assess the general effects of flow cytometric sorting on boar sperm function and IVF outcomes, independent of individual sire variation, allowing baseline characterization of sorting‐associated effects at the population level. This approach allowed detection of sorting‐associated changes in motility subpopulation structure and embryo development, while limiting evaluation of boar‐specific responses to sorting. Whether similar sire‐dependent variability exists in pigs, or whether porcine sperm are more susceptible to sorting‐induced physiological or molecular perturbations, remains unknown and warrants investigation in future studies using individual boars.

Although fertilization was not directly assessed in this study, the interpretation of cleavage outcomes in pigs warrants consideration of polyspermy, which remains a persistent challenge in porcine compared to bovine IVF systems. Bovine embryos have lower polyspermy rates, while porcine oocytes are highly susceptible to polyspermy (Abeydeera and Day 1997). Although polyspermic porcine zygotes can develop to the blastocyst stage, their developmental competence is compromised, as evidenced by reduced inner cell mass cell numbers and post‐implantation growth retardation compared with normally fertilized embryos (Han et al. 1999). Whether the reduced cleavage and blastocyst development observed following fertilization with sorted sperm reflects altered fertilization efficiency, increased polyspermy, or impaired post‐fertilization competence remains unclear. Notably, flow cytometric sperm sorting can induce capacitation‐like biochemical and structural changes in boar sperm, including an approximate 20 percentage‐point increase in the proportion of sperm exhibiting a CTC staining pattern (B pattern; approximately 5% in fresh sperm and approximately 25% following sorting). This is indicative of altered organization and signaling pathways (Bucci et al. 2012) and impaired functional interactions with the female reproductive tract, including reduced sperm binding to oviduct epithelial cells, even when bulk motility measures do not fully explain these functional deficits (Winters et al. 2018).

Analysis of CASA motility parameters revealed that flow cytometric sorting induced statistically significant, yet biologically modest, changes in sperm motility and kinematics. Importantly, these changes did not scale with the pronounced reduction in cleavage and blastocyst development observed following IVF, reinforcing the concept that conventional semen analysis, while useful for describing sperm movement, is a limited predictor of fertilization competence and subsequent embryonic development. In humans, sperm subpopulations with comparable motility profiles have been shown to differ in capacitation status, chromatin integrity, and fertilization potential (Sousa et al. 2011), and studies in boar sperm have reported that bulk CASA metrics remain largely unchanged despite underlying functional alterations (Henning et al. 2013). Previous reports have further highlighted that the impact of sorting on sperm motility is highly variable and species dependent. In pigs, (Großfeld 2007) reported motility differences between sorted and unsorted sperm only after 120 h of storage at 15°C and following incubation at 38°C, whereas another report described reduced motility 24 h post‐sorting (Winters et al. 2018). In contrast, a bovine study demonstrated broader alterations in motility patterns, including reduced proportions of fast and slow motile sperm, decreased hyperactivation, and changes in kinematic parameters such as reduced LIN and WOB, but increased STR and DCL, while others, such as DSL, VSL, VCL, and BCF remained unaffected (Steele et al. 2020). Collectively, these findings underscore that bulk CASA outcomes following sorting may not reliably reflect functional competence across species or experimental conditions. An additional source of cross‐study heterogeneity is the storage and preservation context in which sorting effects have been reported. The present study used fresh, commercially extended boar semen stored at 17°C for ~72 h prior to sorting, whereas published bovine sex‐sorted IVF studies have typically used sorted sperm subjected to cryopreservation and thawing prior to use in IVF. Storage temperature, extender composition, and freeze‐thaw exposure could each independently shape sperm motility‐subpopulation structure and capacitation dynamics. Direct comparison of fresh vs. cryopreserved sorted boar sperm in standardized porcine IVF systems should be assessed in future work.

Consistent with this interpretation, single‐cell analysis revealed that sperm sorting does not generate novel motility phenotypes but instead redistributes existing motility subpopulations. The observed depletion of fast, progressive sperm and enrichment of less energetically demanding motility states following sorting is consistent with prior reports demonstrating that flow cytometric sorting may impact sperm through mechanisms such as mechanical stress, oxidative damage, or mitochondrial perturbation (Balao da Silva et al. 2016; Suh et al. 2005). This interpretation was further supported by t‐SNE, which demonstrated extensive overlap between the sorted and unsorted treatments. Together, these findings indicate that sorting induces subtle but coordinated shifts in the distribution of pre‐existing motility states rather than generating fundamentally altered motility phenotypes, reflecting broader physiological consequences that cannot be solely captured by population‐level CASA motility metrics.

Our recent study (Weide et al. 2026) characterized sorting‐associated effects on boar sperm membrane integrity, acrosomal status, and zinc‐mediated capacitation dynamics, demonstrating that modest changes in those parameters accompany the motility‐subpopulation redistribution reported here. Together, the two studies indicate that sorting compromises porcine IVF outcomes through a combination of subtle physiological perturbations whose individual contributions cannot be resolved at the population level. While the present study provides evidence that flow cytometric sorting compromises porcine IVF outcomes and alters the structure of sperm subpopulations, several mechanistic questions remain. Future studies should aim to quantify the incidence of polyspermy following fertilization with sorted sperm and zona pellucida hardening assessments to determine if sorted sperm alter zona pellucida binding or penetration capability. Additional work evaluating mitochondrial function, reactive oxygen species generation, and potential sire‐dependent variability in sorted sperm could also help clarify the underlying biological mechanisms responsible for reduced in vitro embryo development in pigs.

## 6 Conclusion

Flow cytometric sorting compromised porcine cleavage and embryo development following IVF despite inducing modest changes in sperm motility kinematics. Reductions in cleavage and blastocyst formation following IVF with sorted sperm are generally consistent with trends reported in bovine IVF systems; however, the magnitude of developmental impairment in porcine was greater, with blastocyst development being approximately 10% lower than values typically reported in cattle. Notably, prior porcine sex‐sorting IVF studies reported comparable or higher cleavage rates with sorted sperm; however, those studies used sperm:oocyte ratios 10‐ to 20‐fold lower than standard porcine IVF concentrations due to sorting throughput limitations at the time. These present findings indicate that when sorted sperm are used at standard IVF concentrations, FACS‐based processing impairs porcine cleavage and blastocyst development, and extend prior work by providing a mechanistic link between sorting‐associated redistribution of single‐cell motility subpopulations and embryo developmental competence. Importantly, single‐cell motility analyses revealed that sorting does not generate novel motility phenotypes but instead redistributes existing sperm populations, selectively reducing fast, progressive sperm while enriching less energetically demanding motility states. The observed developmental decline likely arises from subtle physiological or molecular alterations induced during sorting that affect early embryogenesis. Given that polyspermy remains a major obstacle in many porcine IVF systems, future research should determine whether sorting impairs sperm‐oocyte interactions and the establishment of the zona pellucida block to polyspermy.

## Author Contributions

**Isabel Rodriguez:** conceptualization, methodology, data curation, investigation, validation, formal analysis, visualization, writing – original draft, writing – review and editing, funding acquisition. **Tyler Weide:** methodology, data curation, investigation, validation, writing – review and editing. **Caleigh E. Grote:** writing – review and editing, investigation. **Alexandra Keller:** writing – review and editing, investigation. **Catarina Bittencourt:** writing – review and editing, investigation. **Emily Schoelerman:** writing – review and editing, investigation. **Ian Shofner:** investigation, writing – review and editing. **Aileen F. Keating:** supervision, project administration, conceptualization, writing – review and editing, resources. **Bethany K. Redel:** conceptualization, methodology, writing – review and editing, project administration, supervision, resources. **Karl Kerns:** conceptualization, methodology, investigation, funding acquisition, writing – original draft, writing – review and editing, supervision, resources, project administration.

## Ethics Statement

Ethical review and approval were not required for this study. Boar sperm samples obtained in collaboration with industry partners were surplus from routine semen collection for commercial breeding and were not collected specifically for research purposes. Porcine oocytes were recovered from ovaries collected at a local harvest facility as byproducts of standard food production; no animals were slaughtered for the purpose of this study. All sample handling followed established industry and laboratory protocols consistent with ethical and humane treatment of animals. In accordance with institutional and national guidelines, this work was exempt from Iowa State University Institutional Animal Care and Use Committee oversight.

## Conflicts of Interest

The authors declare no conflicts of interest.

## Supporting information

Supporting File

## Data Availability

The data that support the findings of this study are available from the corresponding author upon reasonable request.
