# Supplementary material for: Effects of Fluorescence‐Activated Cell Sorting on Boar Sperm Motility Subpopulations and In Vitro Embryo Development
Source: Mol Reprod Dev. 2026 Jun 7;93(6):e70123. doi: 10.1002/mrd.70123 (PMC13243814; doi:10.1002/mrd.70123)
Supplement: Supplementary file 1 — Supporting File [file MRD-93-e70123-s001.docx]

**Supplemental Material**

| **Parameter** | **Control (unsorted)** | **Sorted** | **FDR-*p*** |
| --- | --- | --- | --- |
| Total Motility | 80.0 ± 2.3^a^ | 59.6 ± 5.6^b^ | 0.0347 |
| Progressive Motility | 74.3 ± 2.9^a^ | 55.8 ± 5.5^b^ | 0.0347 |
| Progressive Circular Motility | 2.7 ± 0.6^a^ | 9.3 ± 2.2^b^ | 0.0453 |

**Supplemental Table 1. General CASA motility parameters of boar sperm by treatment.** Values are presented as mean ± SEM. Different superscript letters within a row indicate significant differences between treatments, as determined by paired t-tests with a Benjamini-Hochberg false discovery rate (FDR) adjustment (FDR < 0.05). This table corresponds to **Figure 2.**

| **Parameter** | **Control (unsorted)** | **Sorted** | **FDR-*p*** |
| --- | --- | --- | --- |
| ALH | 1.2 ± 0.1^a^ | 1.1 ± 0.1^a^ | 0.402 |
| BCF | 17.8 ± 0.8^a^ | 15.8 ± 1.8^a^ | 0.402 |
| DAP | 17.7 ± 0.9^a^ | 19.7 ± 2.3^a^ | 0.462 |
| DCL | 41.6 ± 2.0^a^ | 45.5 ± 4.9^a^ | 0.468 |
| DSL | 11.6 ± 0.6^a^ | 12.1 ± 1.6^a^ | 0.718 |
| HAC | 0.4 ± 0.0^a^ | 0.3 ± 0.0^a^ | 0.402 |
| LIN | 0.3 ± 0.0^a^ | 0.3 ± 0.0^b^ | 0.0347 |
| STR | 0.7 ± 0.0^a^ | 0.7 ± 0.0^b^ | 0.0411 |
| VAP | 55.2 ± 3.4^a^ | 48.9 ± 5.2^a^ | 0.402 |
| VCL | 126.1 ± 7.5^a^ | 112.1 ± 11.1^a^ | 0.402 |
| VSL | 40.2 ± 2.3^a^ | 32.5 ± 3.9^a^ | 0.135 |
| WOB | 0.4 ± 0.0^a^ | 0.4 ± 0.0^a^ | 0.669 |

**Supplemental Table 2. Detailed CASA motility parameters of boar sperm by treatment.** Values are presented as mean ± SEM. Different superscript letters within a row indicate significant differences between treatments, as determined by paired t-tests with a Benjamini-Hochberg false discovery rate (FDR) adjustment (FDR < 0.05). This table corresponds to **Figure 3.**


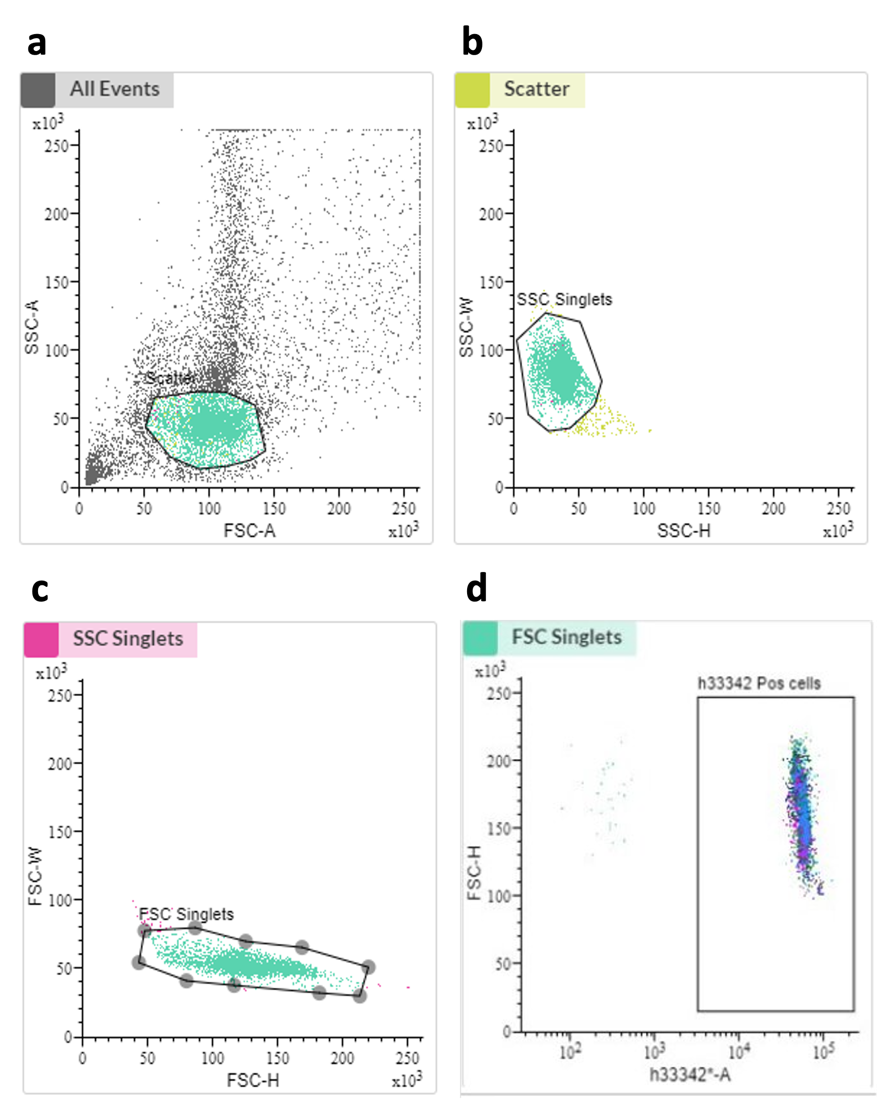


**Supplemental Figure 1. Sequential gating approach for isolating sperm singlets. a**) Forward scatter versus side scatter gating was used to visualize all detected events and to define the main sperm population (FSC-A vs SSC-A), with aggregates and debris excluded based on light scatter characteristics. **b**) Identification of singlets using side scatter width (SSC-W) versus height (SSC-H). **c**) Confirmation of singlet population using forward scatter width (FSC-W) versus height (FSC-H). **d**) Final gating of Hoechst 33342-positive (H33342-A) sperm nuclei within the FSC singlet population.


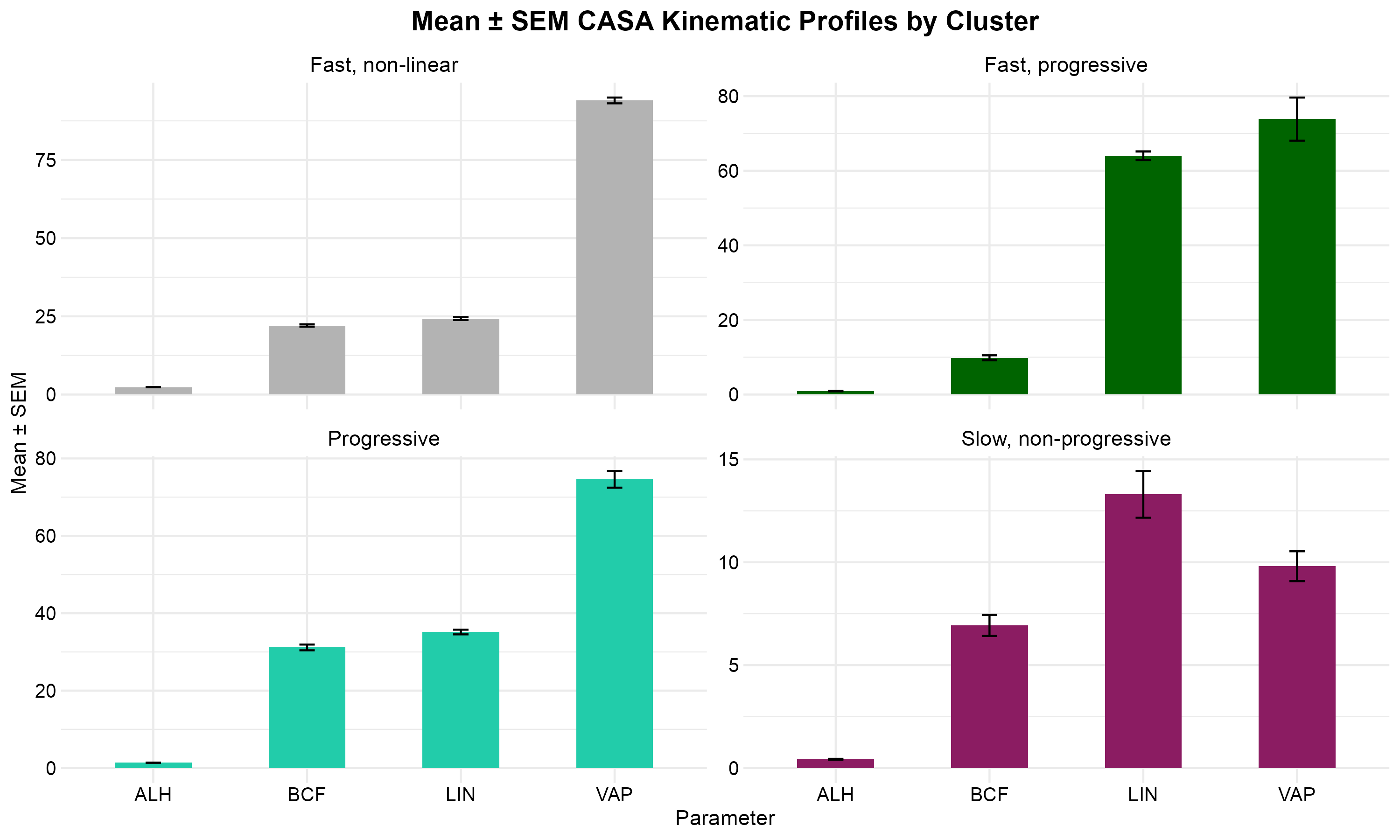


**Supplemental Figure 2. Cluster-specific CASA kinematic profiles.** Mean (± SEM) CASA parameters (VAP, LIN, ALH, and BCF) were calculated at the replicate level (n = 10) for each cluster and used to define biologically meaningful motility subpopulations based on relative velocity and linearity profiles.


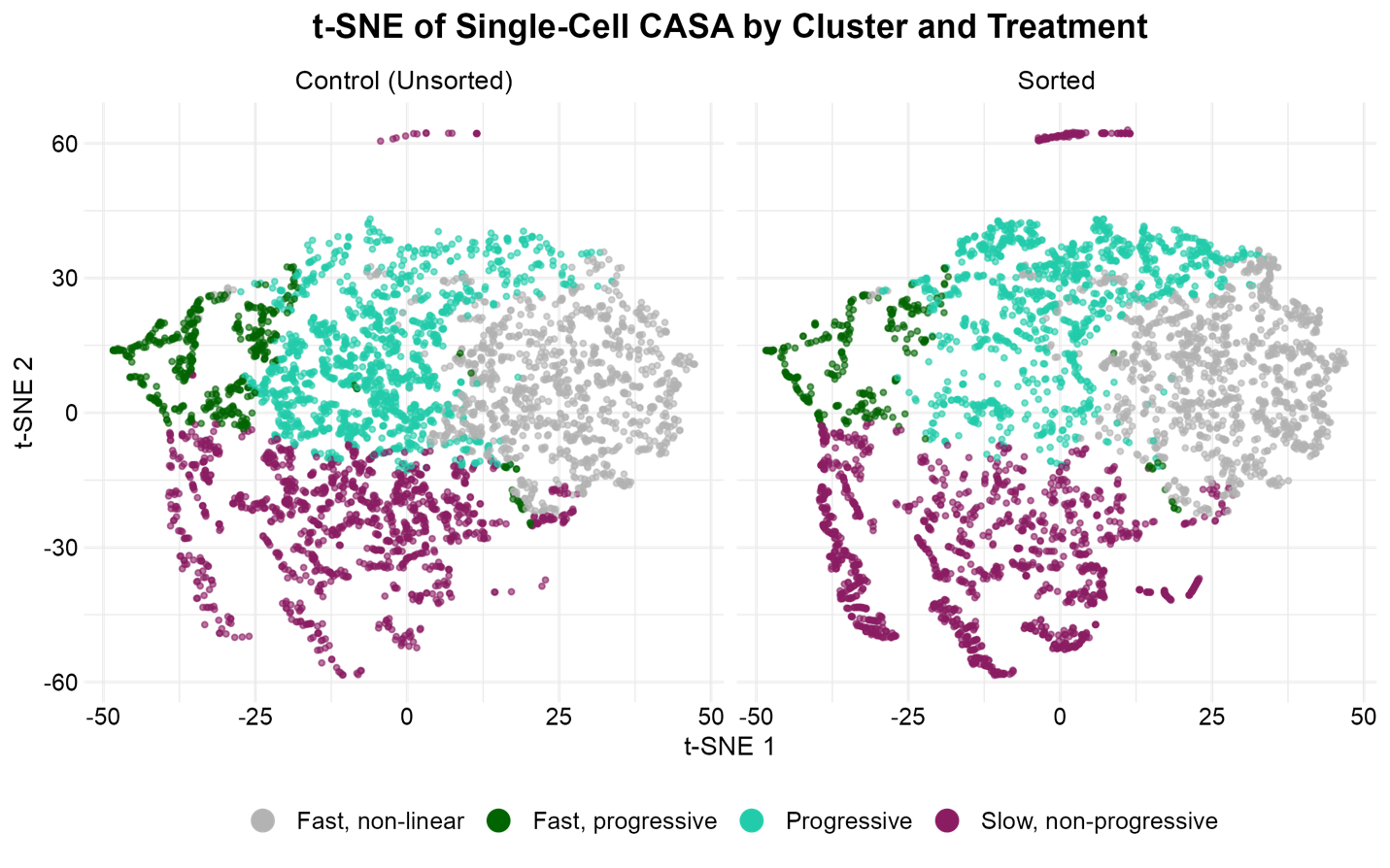


**Supplemental Figure 3. t-SNE visualization of single-cell CASA kinematics by cluster and treatment.** t-distributed stochastic neighbor embedding (t-SNE) was performed on single-cell CASA kinematic variables (VAP, LIN, ALH, and BCF) to visualize sperm motility phenotypes. Clusters were generated using unsupervised k-means (k = 4). Points represent individual sperm cells and are colored by cluster assignment, with panels faceted by treatment.
